# Plasmatic Exosome Number and Size Distinguish Prostate Cancer Patients From Healthy Individuals: A Prospective Clinical Study

**DOI:** 10.3389/fonc.2021.727317

**Published:** 2021-10-20

**Authors:** Mariantonia Logozzi, Davide Mizzoni, Rossella Di Raimo, Alessandro Giuliani, Martina Maggi, Alessandro Sciarra, Stefano Fais

**Affiliations:** ^1^ Department of Oncology and Molecular Medicine, Istituto Superiore di Sanità, Rome, Italy; ^2^ Environment and Health Department, Istituto Superiore di Sanità, Rome, Italy; ^3^ Department of Urology, Policlinico Umberto I, Università La Sapienza, Rome, Italy

**Keywords:** plasmatic exosomes, prostate cancer, NTA, screening test, liquid biopsy

## Abstract

There is a urgent need for valuable strategy in early and less invasive diagnosis for cancer. Preliminary data have shown that the plasmatic levels of exosomes increase in cancer condition. This study investigates the relevance of plasmatic levels and size distribution of exosomes in 42 individuals with no signs of urological disease (CTR) as compared to 65 prostate cancer patients (PCa). It was used Nanoparticle Tracking Analysis (NTA), a highly reliable and sensitive method for exosomes characterization and quantification. The relation structure among the NTA-derived parameters was assessed by means of Principal Component Analysis, which allowed detecting the global discriminant power of NTA test in terms of Receiver Operating Characteristic (ROC) curve and the selection of cut-off thresholds. The results showed that PCa had significantly higher plasmatic levels of exosomes and that the exosomes were smaller in size as compared to the CTR; the values reached 89% sensitivity and 71% specificity, in distinguishing PCa from CTR. These results propose a new exosome-based non-invasive clinical approach for the clinical follow-up of prostate cancer undergoing surgical treatment; in addition this method may be developed as a new screening test for prostate cancer’s early diagnosis. While this clinical study was performed in prostate cancer, it may represent a proof of concept extendable to virtually all cancers, as it is suggested by both pre-clinical evidence and clinical data obtained with different technical approaches.

## Introduction

Among the common phenotypes characterizing malignant tumors, hypoxia, low nutrient supply, extracellular acidosis are by far the most relevant. Recent evidence has suggested that the increased number of exosomes may well implement this list (1–4). Exosomes received considerable attention in the last decade for their peculiar structure, biophysical properties and function in a plethora of biological processes in which they are involved (5–7). Exosomes are extracellular nanovesicles (40–180 nm) released by virtually all cell types under normal and pathological conditions (5, 6, 8–10). Thanks to the ability to transmit their cargo of lipids, proteins, DNAs, mRNAs, miRNAs, and other metabolites into the target cells, exosomes play a pivotal role in intercellular communication. Indeed, exosomes can modulate both physiological and pathological processes, including tumor progression, elimination of toxic substances, as well as drug and therapeutic antibodies delivery (5, 6, 8, 10–15). For these reasons they have been investigated for a clinical application as well, including both diagnosis and therapy.

In fact, due to their ability to deliver a broad range of molecules, exosomes are considered the ideal source of new and more specific tumor biomarkers (5–7, 16–26), including fully active molecules (e.g. CAIX) (27). The few clinical studies have shown that exosomes are detectable in many biological fluids where they have been investigated in both normal and disease conditions (5, 28–31). Exosomes continuously travel the body and an interest is growing to their ability to protect delivered molecules by packaging them within lipid vesicles (32).

However, to date, notwithstanding the increasing preclinical evidence, the data supporting the presence of specific tumor markers in exosomes from either plasma or other body fluids samples are still inconclusive. On the other hand, a critical role of plasmatic levels of exosomes in the clinical follow up of tumor patients has been hypothesized (18). The first evidence supporting the potential use of exosome levels in human body fluids as a tumor progression marker was in melanoma patients with advanced disease (28). Melanoma patients showed significantly increased level of plasmatic exosomes as compared to healthy donors (28). Pre-clinical evidence has also shown that the increased levels of plasmatic exosomes were directly related to the presence of a tumor mass (28). More recent reports have shown that the surgical treatment of the primary tumor led to a dramatic reduction of the plasmatic exosome levels (33, 34). Preclinical investigation has shown that the microenvironmental acidity induces a marked increase in exosome release by tumor cells, independently from the tumor histotype, thus providing a possible etiopathogenetic role of paracrine factors for the increased plasmatic levels observed in cancer patients (9, 35). Pre-clinical investigation has also shown that tumor microenvironmental acidity is responsible for the release of smaller exosomes with a more homogeneous distribution as compared to the exosomes released at buffered conditions (9, 35). Thus, it appears conceivable to hypothesize that in tumor patients microenvironmental acidity may have a pivotal role in determining both the increase of circulating exosomes and their size reduction (10). Together with influencing exosome number and size, tumor acidity induces over-expression of known tumor biomarkers such as PSA (Prostate Specific Antigen) in exosome from prostate cancer patients (35). A recent clinical study has shown that the expression of PSA on plasmatic exosomes distinguished prostate cancer patients from both Benign Prostate Hyperplasia (BPH) and healthy subject (36). Tumor microenvironmental acidity influenced also the expression of proteins, such as CAIX, that on exosomes exert their full enzymatic activity (27, 37). Moreover, CA IX expression and activity were correlated to the exosome intraluminal acidic pH, showing for the first time that plasmatic exosomes from tumor patients are acidic (27).

Some clinical studies have also shown that the number of plasmatic exosomes may represent a valuable new tool for monitoring cancer patients, while obtained with two different techniques and in different cancer hystotypes, such as prostate cancer (35) and oral cancers (34). On the basis of these two very preliminary studies we decided to carry on with a clinical study aimed at assessing the clinical relevance of both the number and size distribution of plasmatic exosomes independently from the potential presence of known or unknown molecular biomarkers. To this purpose we compared a cohort of prostate cancer patients (PCa), to individuals with no signs of urological disease (CTR). The technological approach was to exploit the Nanoparticle Tracking Analysis (NTA), following the repeated rounds of ultracentrifugation, for exosomes characterization and quantification since it is considered a reliable, efficient, and objective technique for the study of exosomes (9, 28, 35, 36, 38–40). This assay was performed in plasma samples from 65 PCa and 42 CTR, providing detailed information on both the exosomes plasmatic levels (particles/ml) and the size distribution (nm). The results showed that the number and size of plasmatic exosomes significantly distinguished PCa patients from the CTR group with high sensitivity and specificity. We consider our results of great importance in providing a non-invasive new tool allowing to distinguish prostate cancer patients from healthy subjects, but also exploitable for early screening, diagnosis, and clinical follow-up of all malignant tumors.

## Materials and Methods

### Population

The review board of each participating institution approved the trial, which was conducted in accordance with the current International Conference on Harmonisation guidelines for Good Clinical Practice and the principles of the Declaration of Helsinki. The study was approved by the Istituto Superiore di Sanità Ethics Committee on 18/04/2017 (Rif. Prot. PRE-275/17). Written informed consent was obtained from all subjects involved in the study.

All authors assume responsibility for the completeness and accuracy of the data and analyses and for the fidelity of the trial to the protocol. All the authors had full access to the data, drafted the manuscript, reviewed and approved the manuscript before submission, and made the decision to submit the manuscript for publication. No sponsor provided funding for the study.

Eligible cases were divided in 2 groups: control cases (CTR) and prostate cancer cases (PCa). All cases were consecutively included in the study as out-patients referred to Department of Urology on the basis of the inclusion criteria. Patients were correctly informed, accepted to be included in the study, and signed an informed consensus prior to each procedure. Human plasma samples were collected from EDTA-treated whole blood, 5 mL into BD Vacutainer^®^ K3-EDTA-coated collection tubes (Beckton Dickinson, Franklin Lakes, NJ, USA), from department of Urological Sciences, Policlinico Umberto I, Sapienza University of Rome, Italy. Once collected, the samples were labeled by the clinical center with an identification code and were manipulated anonymously and blinded in the testing phase with the code assigned by the clinical center.

This is an experimental observational clinical research study in which no additional and/or administered drug tests and/or modified therapy are performed. The aim of this study was to compare a population of males without signs of urological disease to prostate cancer patients that were pooled between individuals with different Gleason score.

More in details:

**CTR.** The control group consisted of 42 male individuals consecutively referred to our department with the following inclusion criteria: age from 18 to 50 years; no clinical evidence of BPH or PCa [digital rectal examination (DRE) and ultrasonography (US)]; prostate volume less than 30 cc; total PSA level less than 1.4 ng/mL; no familiarity for PCa; no therapies that can influence PSA determination; no acute prostatic inflammation; no prostatitis.
**PCa.** The PCa group consisted of 65 male individuals from 51 to 80 years consecutively referred to our department with a histologically confirmed diagnosis of prostate adenocarcinoma (prostate biopsy). Total PSA (ng/mL) were from 1.8 to 100.0. None of cases was submitted to androgen deprivation therapies, chemotherapies, new generation hormone therapies or other therapies that can influence PSA determination. No acute prostatic inflammation, no prostatitis. All cases were stratified in risk classes (low, intermediate or high according to EAU classification) based on total PSA levels, Gleason score [6 (3 + 3), 7 (3 + 4), 7 (4 + 3), 8-10], and clinical stage (T1-T2 N0 M0, T3 N0 M0 or N1).

### Preparation of Exosomes From Plasma of CTR and PCa

To obtain plasma from blood samples, EDTA-treated blood from PCa patients and CTR were centrifuged at 400 x g for 20 min. Plasma was then collected and stored at −80°C until analysis. Upon thawing, 1 mL of plasma underwent the centrifugal procedure as previously described (6, 41) in order to eliminate cell debris, organelles and microvesicles, and pellet exosomes. In the last step, plasma samples were centrifuged for 1 h 30 min at 110,000 x g using a Fiberlite™ F50L-24 x 1.5 Fixed-Angle Rotor, K-Factor: 33 (Thermo Fisher Scientific, Waltham, MA, USA) in the Sorvall WX Ultracentrifuge Series (Thermo Fisher Scientific), to obtain the exosomal pellet, which was then washed in PBS and resuspended in the appropriate buffer for subsequent analyzes. In particular, the exosomal pellet was resuspended in PBS for Nanoparticle Tracking Analysis and Flow Cytometry Analysis, and in CHAPS buffer 1x for western blot analysis.

### Nanoparticle Tracking Analysis

Nanoparticle Tracking Analysis (NTA) from Malvern (NanoSight NS300, Worcestershire, UK) was used for the measurement of size distribution and concentration of exosomes samples in liquid suspension in the range from 10 – 1000 nm based on the analysis of Brownian motion (35). Following laser beam illumination, the light scattering allowed to visualize, record and track the particles with a CCD or CMOS camera. Five videos of typically 60 s duration were taken. Data were analyzed using the NTA 3.0 software (Malvern Instruments) which was optimized to first detect and then track each particle on a frame-by-frame basis. NTA is based on the phenomenon of the random movement (diffusion) of small particles when they are dispersed in a liquid, allowing direct and precise measurement of the concentration and size of the particles. The Brownian motion of each particle was tracked using the Stokes–Einstein equation: D° = kT/6πηr, where D° is the diffusion coefficient, kT/6πηr = f_0_ is the frictional coefficient of the particle, for the special case of a spherical particle of radius r moving with uniform velocity in a continuous fluid of viscosity η, k is Boltzmann’s constant, and T is the absolute temperature.

The evaluation of the Particle Size Distribution (PSD) was performed through the parameters Mean, Mode, SD, D10, D50 (Median) and D90 which indicate respectively the average, most frequent particle class size, standard deviation, and the 10%, 50% and 90% percentiles of the analyzed particles. Specifically, D10, D50 and D90 indicate the size below which 10%, 50% and 90% respectively of total number of exosomes is included, mean and mode point to the average particle size and the most represented size value respectively, while SD is the standard deviation (average distance from the mean) of the distribution.

### Western Blot Analysis

For the two groups (CTR and PCa), 4 mL of plasma was pooled and Size Exclusion Chromatography (SEC) was performed for the isolation of plasma-derived exosomes, as described previously (42).

Exosomes from plasma of CTR and PCa patients were lysed in CHAPS buffer 1x containing Tris 10 mM pH 7.4, MgCl_2_ 1 mM, ethyleneglycoltetraacetic acid (EGTA) 1 mM, CHAPS 0.5%, glycerol 10%, phenylmethylsulfonyl fluoride (PMSF) 1 mM and protease inhibitor cocktail (1 µg/mL leupeptin, 1 µg/mL pepstatin A, 1 µg/mL aprotinin, and PMSF 1 mM). Protein concentration was determined using the Bradford protein assay (Bio-Rad Laboratories, Inc, Hercules, CA, USA). Thirty micrograms of exosomal lysates were resolved on 10% acrylamide gel and transferred to a Protran BA85 nitrocellulose membrane (Schleicher & Schuell, Keene, NH, USA).

Nonspecific binding sites were blocked by incubation in PBS containing 0.05% Tween 20 and 5% milk powder. Blotting was performed using anti-Tsg 101 (C-2, Santa CruzBiotechnology, Dallas, TX, USA), anti-CD81 (B-11, Santa Cruz Biotechnology, Dallas, TX, USA), and anti-Alix (3A9, Thermo Fisher Scientific, Waltham, MA, USA) monoclonal antibodies, for 18 h at 4°C. After incubation with appropriate anti-mouse peroxidase-conjugated secondary antibody (IgG; Amersham Biosciences, Milan, Italy) for 1 h at room temperature, membranes were revealed by enhanced chemiluminescent (ECL) substrate (Thermo Fisher Scientific,Waltham, MA, USA).

### Flow Cytometry Analysis of Exosomes

Exosomes purified from plasma were diluted in PBS in a final volume of 50 µL. Anti-human CD81 allophycocyanin (APC) conjugated (Beckman Coulter, Brea, CA, USA) and anti-human PSA fluorescein (FITC) conjugated (clone 5A6, Abcam, Cambridge, UK) or anti-human IgG2a APC conjugated and anti-human IgG1 FITC conjugated (Beckman Coulter) were added to the exosome preparation at optimal pre-titered concentrations and left for 20 min at RT. The same procedure was performed for the analysis of anti-human CD9 phycoerythrin (PE) conjugated (M-L13, RUO (GMP) BD Biosciences, USA) and anti-human PSA fluorescein (FITC) conjugated (clone 5A6, Abcam, Cambridge, UK), using anti-human IgG1 (PE) conjugated and anti-human IgG2a (FITC) conjugated as isotype controls, respectively.

500 µL of PBS were added to samples before the acquisition on the CytoFLEX flow cytometer (Beckman Coulter).

The cytometer was calibrated using a mixture of non-fluorescent silica beads and fluorescent (green) latex beads with sizes ranging from 110 nm to 1300 nm. This calibration step enables the determination of the sensitivity and resolution of the flow cytometer (fluorescent latex beads) and the size of extracellular vesicles (silica beads). All samples were acquired at low flow rate for the same amount of time in order to obtain an estimate of absolute counts of exosomes comparable between various samples. The analysis of the data was performed with FlowJo software (FlowJo, LLC; Ashland, Oregon, USA) (35, 36).

### Statistical Analysis

The inferential statistics was based upon the t-test over the above described parameters of PSD distribution, adopting the Satterwaithe correction when in presence of a statistically significant difference in the standard deviation of the two groups.

The relation structure among the NTA-derived parameters was assessed by means of Principal Component Analysis: the first two extracted components (PCnano1, PCnano2) were used to calculate a canonical variate by which assess the global discriminant power of NTA test in terms of Receiver Operating Characteristic (ROC) curve. ROC strategy allowed the estimation of both the global discriminant ability of the test (area under the roc curve, AUROC) and the selection of cut-off thresholds maximizing sensitivity (percentage of correctly diagnosed PCa patients) and specificity (percentage of negative result in CTR individuals) (43). In order to eliminate the suspect of an effect of disease/age necessary link, we checked the possible confounding role of different ages in the two groups by computing the Pearson correlation between both exosome concentration and size with age separately in the two CTR and PCa classes. No statistically significant correlation was scored (**Supplementary Table S1**). The lack of any statistically significant correlation between age and exosome descriptors, albeit indirectly, rules out any possible effect of age on the results. The statistical analysis of the results obtained was being performed with the SAS System program 9.4 version. The analysis of the ROC curves was performed by Sigma Plot 11.2 version.

## Results

### Characterization and Distribution of Plasma Exosomes Between PCa Patients and Individuals With No Signs of Urological Disease (CTR) by Nanoparticle Tracking Analysis (NTA)

**Figure 1:**
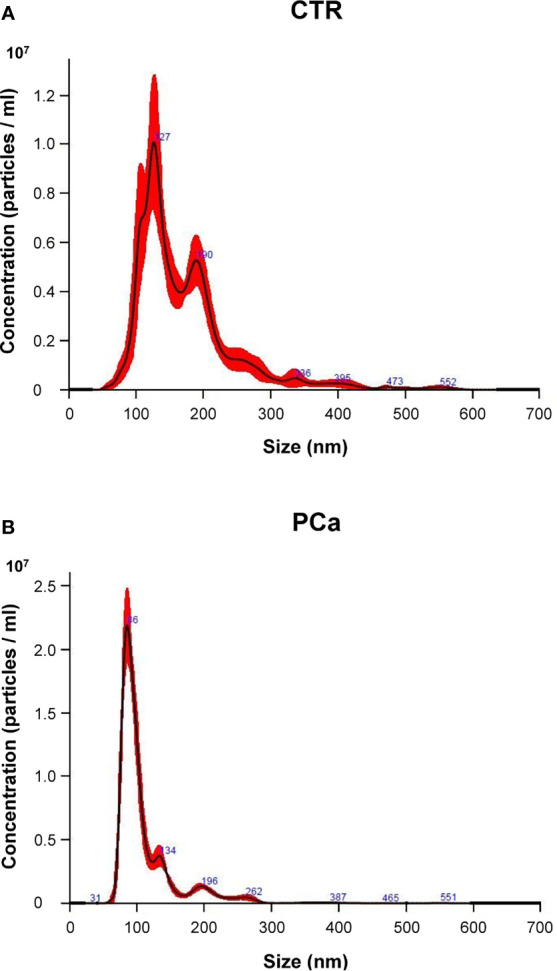
Representative NTA distribution of plasma exosomes in CTR individuals **(A)** and PCa patients **(B)**.

Plasma exosomes from PCa and CTR were characterized for number and size distribution by NTA (**Figure 1**), for the expression of exosome housekeeping markers by Western blot analysis (CD81, Tsg 101 and Alix, **Supplementary Figure S1**) (35, 36), and for the contemporary expression of exosome housekeeping markers (CD9 and CD81) and PSA by Nanoscale Flow Cytometry (**Supplementary Figure S2**) (35, 36).

We first compared PCa and CTR groups in terms of both size and number (concentration) by NTA (**Figure 1**). **Figure 1** shows a representative NTA distribution of exosome samples by either CTR (**Figure 1A**) or PCa (**Figure 1B**), as far as either size (nm, abscissa axis) or number (particles/ml, ordinate axis) are concerned.

The statistical analysis showed a significant difference between CTR and PCa exosome plasma samples (**Table 1**) for both the concentration and the size parameters (with the only exception of SD). In particular the difference in terms of the exosome number between PCa patients and CTR was highly significant (p<0.0001).

**Table 1:** Descriptive and inferential statistics or the two patient groups (PCa = Prostate Cancer, CTR = individuals with no signs of urological disease) as for the entire set of NTA derived descriptors.

Variable	Group	Mean	Std. Dev.	p (t-test)
***Number (concentration*,** ***particles/mL)***	PCa	2.88 x 10^9^	1.43 x 10^9^	<0.0001
	CTR	1.56 x 10^9^	0.57 x 10^9^	
***Mean (size, nm)***	PCa	131.4	21.44	<0.0005
	CTR	145.9	18.82	
***Mode (size, nm)***	PCa	89.53	13.77	<0.003
	CTR	97.44	12.26	
***D10 (size, nm)***	PCa	80.02	10.99	<0.0001
	CTR	88.10	9.09	
***D50 (size, nm)***	PCa	109.8	19.06	<0.0001
	CTR	124.6	18.01	
***D90 (size, nm)***	PCa	210.96	35.96	0.0081
	CTR	228.57	30.77	
***SD (size variability, nm)***	PCa	64.42	11.70	NS
	CTR	68.05	10.16	

NS, Not Significant.

It is worth noting a significant increase in number of exosomes in PCa as well as the shrinking in their size as registered by all the size descriptors. On the other hand, the SD of the size distributions relative to PCa and CTR are substantially identical, suggesting a general (‘rigid’) shift of distribution going from CTR to PCa.

**Figure 2:**
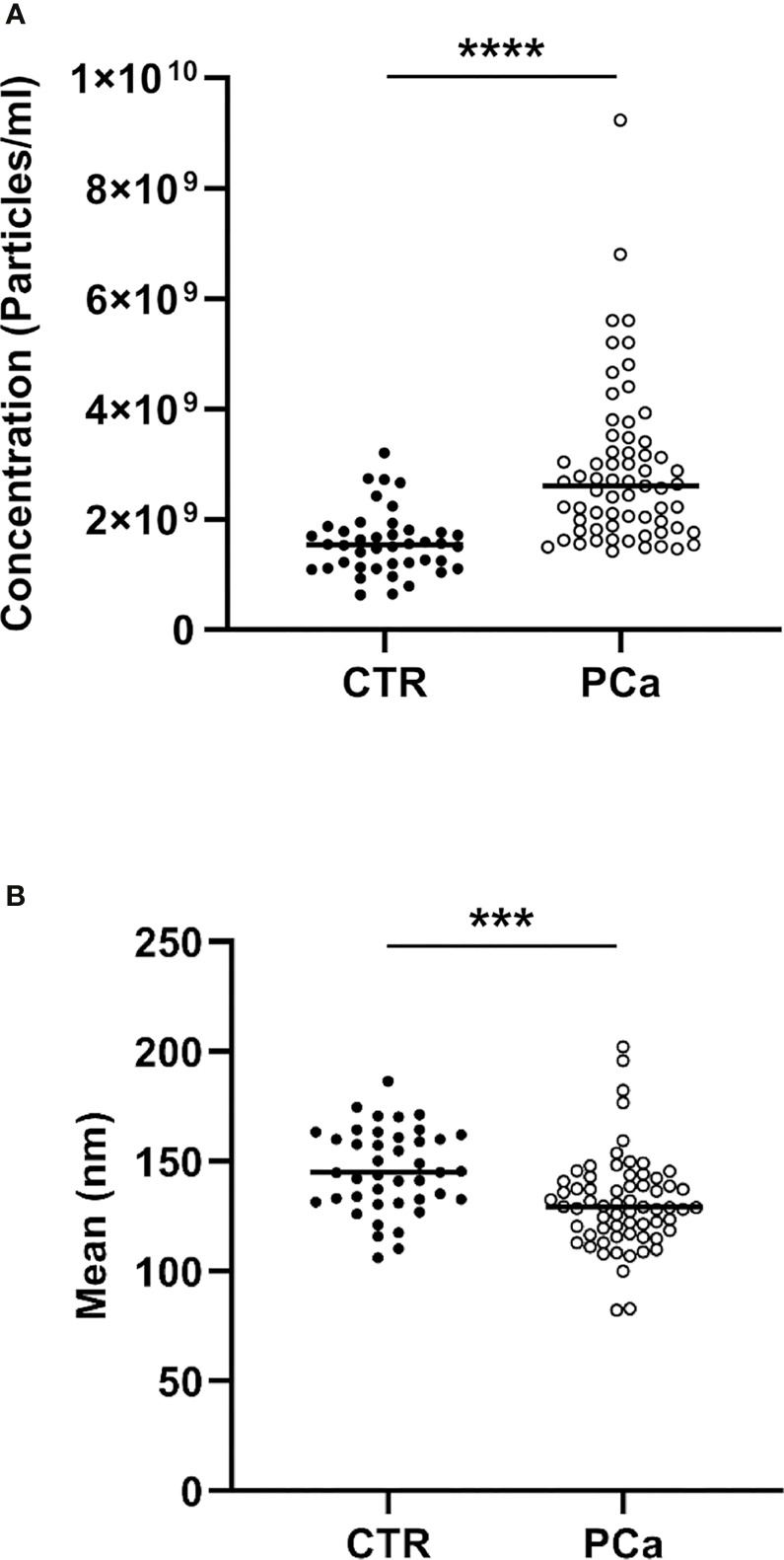
NTA distribution and quantification of CTR and PCa plasmatic exosomes by concentration **(A)** and size **(B)** parameters included within the 25th and 75th percentiles.***p< 0.001, ****p< 0.0001.

**Figure 3:**
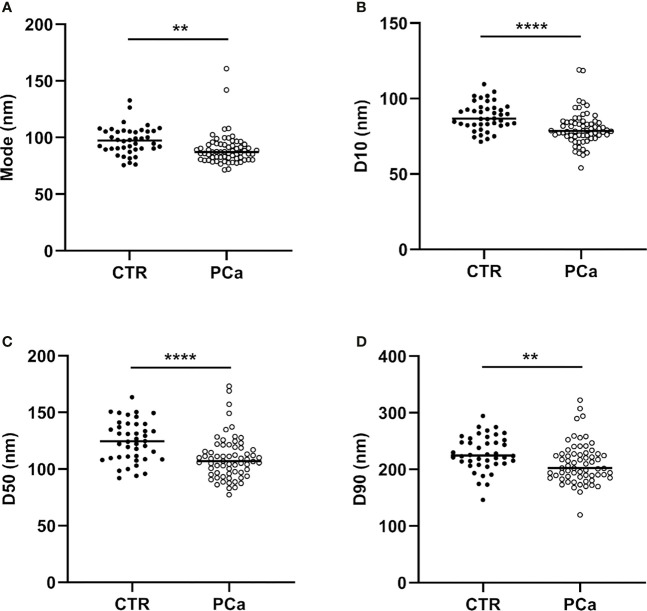
NTA distribution parameters (mode, D10, D50 and D90), correspondent to **(A)**, **(B)**, **(C)** and **(D)** panels respectively of CTR and PCa plasmatic exosomes included within the 25th and 75th percentiles. **p < 0.01, ****p< 0.0001.

In detail, the graphs in **Figures 2** and **3** represent the NTA variables distribution of PCa and CTR included within the 25th and 75th percentiles, discriminating PCa from CTR. PCa exosomes were not only more numerous, but also smaller than the CTR exosomes. In fact, all the dimensional distribution parameters analyzed (such as mean, mode, D10, D50 and D90) were significantly different between the two groups (**Table 1**; **Figures 2** and **3**). Mean (nm) and mode (nm) are parameters useful for describing the set of size of exosomes and their frequency distribution in each plasma sample. D10, D50 and D90 are dimensional parameter that indicate that 10%, 50% and 90% respectively of the exosomes are included below the corresponding nanometers, indicating the spread of exosomes sizes within the sample.

After, the number of PSA-expressing exosomes (NSFC-exo) was acquired using the Nanoscale Flow Cytometry (NSFC) technique and underwent the same statistical analysis adopted for assessing the between group differences relative to the global exosome population. Interestingly, the PSA-specific index (NSFC-exo) showed an almost perfect separation between the groups for the virtual absence of PSA-carrying nanoparticles in healthy subjects (data not shown), supporting our previous results (36).

The aim of the current study was primarily to compare the NSFC-exo in terms of exosomes number and size, between PCa patients and CTR. The analysis of PSA-specific index must be intended as instrumental for checking the hypothesis if the aspecific exosome distribution approach was guided by PSA-containing sub-population of vesicles or if it carried autonomous information as we discuss in the following.

### Mutual Relation Between the Size and Number of PCa and CTR Plasma Exosomes

The following statistical analysis was aimed at investigating the mutual relationships between size and number of plasmatic exosomes. For this purpose, we computed a Principal Component Analysis (PCA) over the original data set having the participants (both CTR and PCa) as statistical units and the NTA-derived indexes as variables (**Table 2**). Three principal components explain the totality of the variance (96.7%), but the first two components (PCnano1, PCnano2) account for the by far the most relevant part of information. As a matter of fact the relative proportion of variance explained by the two main components was: PC1 (PCnano1): 71.7%, PC2 (PCnano2) = 15.0% with a cumulative proportion of explained variance equal to 86.7%.

**Table 2:** Component Loadings.

Loading pattern			
Variable	PCnano1	PCnano2	PCnano3
***Concentration***	-0.00776	**0.97176**	0.22901
***Mean***	**0.99266**	-0.02747	-0.04256
***Mode***	**0.86521**	-0.01709	0.41626
***SD***	0.77564	0.23181	-0.56827
***D10***	**0.92206**	-0.15468	0.28347
***D50***	**0.96473**	-0.10536	0.10432
***D90***	**0.94959**	0.12012	-0.24998

The bold values correspond to the original variables with higher correlation with extracted components (Component Loading).

**Figure 4:**
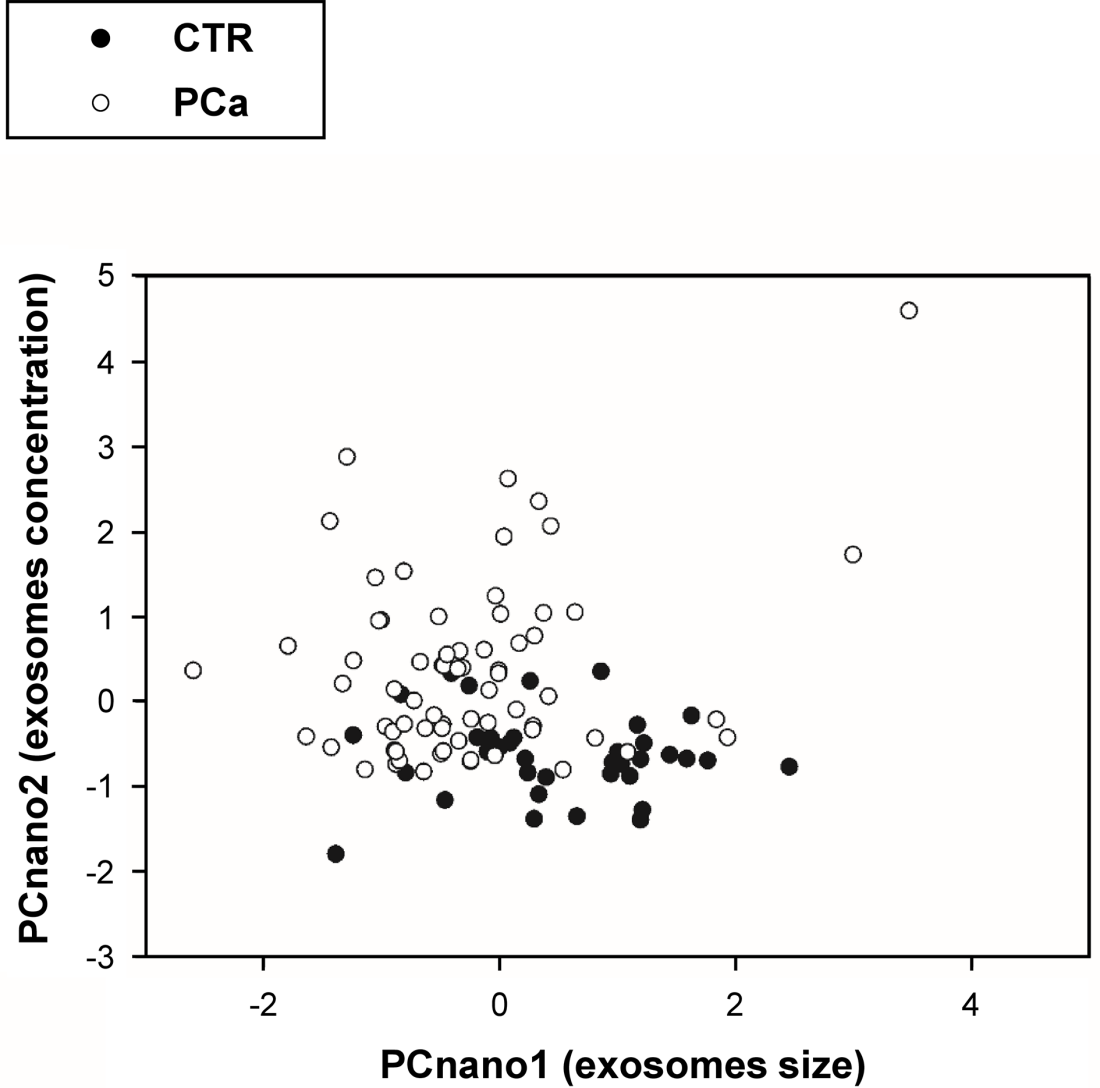
Projection (component scores) of participants in the bi-dimensional space spanned by the two principal components (PCnano1 = exosome size component, and PCnano2 = exosomes number component).

The inspection of the loading matrix (loadings are the correlation coefficients between original variables and components, bolded the most relevant correlations) is reported in **Table 2** allows us to immediately discover the mutual independence of size (PCnano1) and number (PCnano2) of exosomes (principal components are each other orthogonal by construction). Both size (PCnano1) and concentration (PCnano2) allow for a clear separation of PCa and CTR patients (**Figure 4**).

Assuming that the principal components are each other orthogonal by construction, the mutual independence of the size and number of exosomes suggests that the PCa *vs*. CTR separation obtained by these two components results from two independent mechanisms even if both related to cancer condition. This is evident in **Table 3** reporting the descriptive and inferential statistics for PCnano1 and PCnano3 in the two groups. It is worth noting that principal component scores have by construction zero mean and unit standard deviation on the entire data set (PCa + CTR) and are each other mutually orthogonal. Both size and concentration components show a neat statistical significance as for PCa *vs* CTR comparison, their mutual linear independence allows us to hypothesize that shrinkage and concentration increase of exosomes derive from two different mechanisms, even if both related to cancer condition.

**Table 3:** Descriptive and inferential statistics of the two patient groups (PCa and CTR) as for the two main principal components of NTA derived descriptors.

Variable	Group	Mean	Std. Dev.	P (t-test)
***PCnano1*** ***(size)***	PCa	-0.258	1.00	<0.0007
	CTR	0.400	0.870	
***PCnano2*** ***(concentration)***	PCa	0.390	1.05	<0.0001
	CTR	-0.604	0.490	

The plot in **Figure 4** reports the distribution of exosomes in the PCnano1 (size) and PCnano2 (concentration), highlighting a clear shift of tumor samples on the top left part of the graph (high number/small size).

### ROC Curve Between PCa Patients and CTR

While the above reported analyses re-assure us of the biological relevance of both exosome concentration and size in cancer, they do not allow to assess the prognostic and diagnostic relevance of ‘aspecific’ exosome descriptors. In a previous study (36) we already assessed the ability of a specific (PSA-carrying) exosome sub-population in discriminating prostate cancer from healthy donors. The results of the above study actually provided a new approach in distinguishing not only prostate cancer from individuals without a cancer, but also prostate cancer patients from patients with prostate benign hypertrophy (BPH), that is considered a benign inflammatory condition, but with some signs that too often may lead to a cancer over diagnosis, such as the serum PSA levels. However, we were also very curious to extend our previous very preliminary observation showing higher levels of plasmatic exosomes in prostate cancer patients, while with small numbers. With the results of the present study we do not want to suggest a generalized shift from a specific to an aspecific approach; rather a complementary use of the aspecific approach (i.e. plasmatic exosome levels) in a ‘primary screening for the presence of a cancer disease’ made by the Nanoparticle Tracking Analysis technology that can provide a precise analysis of both number and size of plasmatic exosome, while with no direct relation to their specific content, in terms of molecular biomarkers.

Here we pursue a much more ambitious goal: to use only exosome-related information with no reference to a specific biomarker for cancer screening. As a consequence, we do expect a decreased predictive power with respect to the specific approach. This decrease in predictive power is in any case balanced by the much easier (and less costly) procedure and by the promise the simple evaluation of exosome concentration and size could be a warning signal of the presence of a cancer, independently of its particular biotype. We faced this task by a canonical discriminant analysis having as X variables PCnano1 and PCnano2 and as Y variable the healthy/patient categorization. The goal of canonical analysis is to generate a pair (canonical variates) of linear combinations of X and Y variables endowed with maximal mutual correlation (44). In the particular case of canonical discriminant analysis there is only one Y variable expressed in categorical (in this case binary) values. Thus implies we are looking for the linear combination of PCnano1 and PCnano2 that allows for the best separation of PCa and CTR subjects. The procedure generated a pair of canonical variates endowed with a statistically significant correlation (Canonical Correlation = 0.58, F-value = 27.05 p < 0.0001). The formula of linear combination of the canonical variate relative to PCnano1 and PCnano2 was CanVar1 = -0.68 *PCnano1 + 1.02*PCnano2.

**Figure 5:**
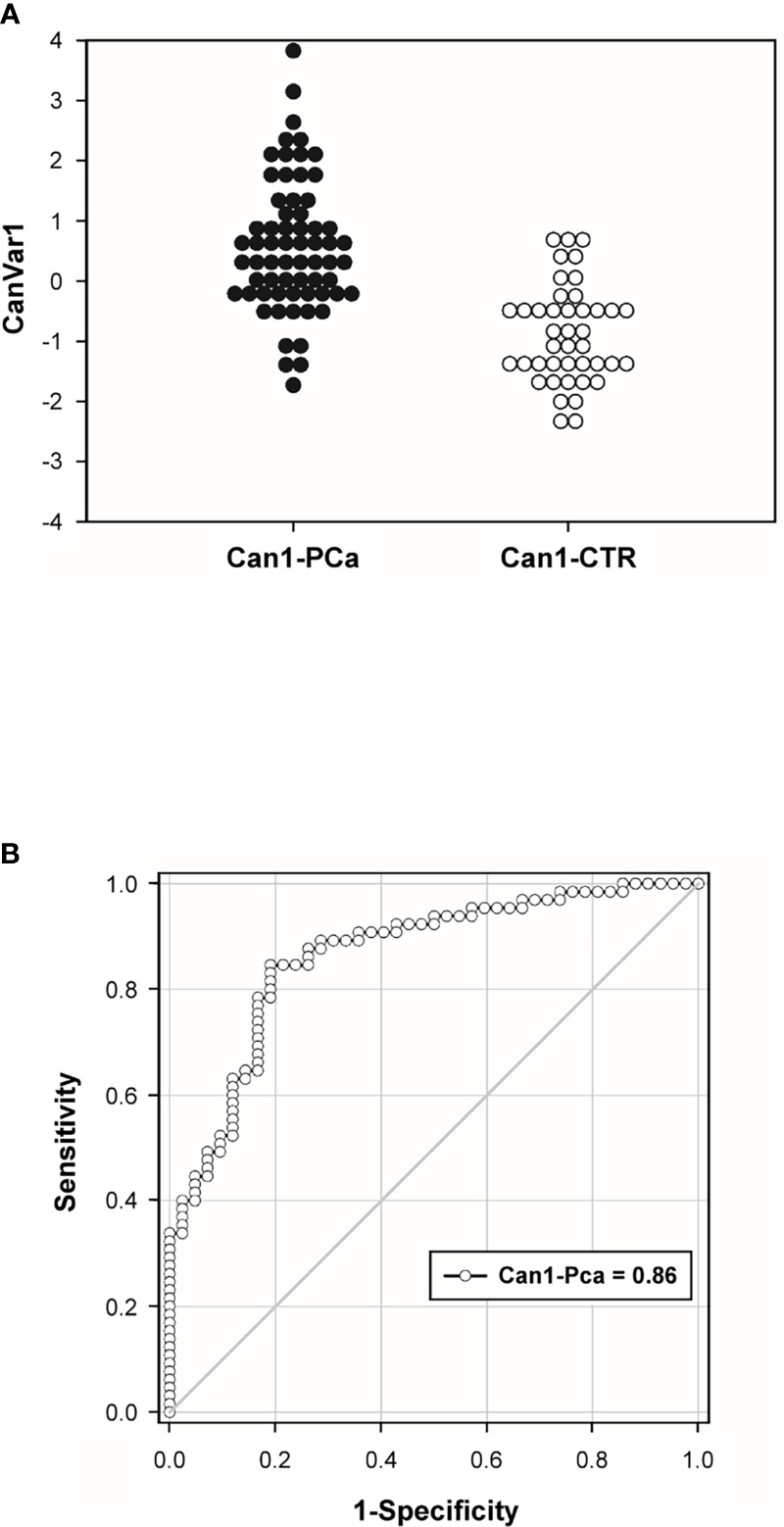
Canonical Variate distribution (CanVar1) **(A)** and Receiving Operating Characteristics (ROC) curve **(B)** relative to cancer-healthy discrimination based on CanVar1.

As expected, the coefficient for the number of exosomes component (PCnano2) was higher than the one for exosomes size due to the higher discriminant ability of exosome number with respect to their size. Under the same heading, the coefficients for the two components have an opposite sign reminiscent of the ‘increase in number’ and ‘decrease in size’ effect of cancer (**Figure 5A**).

The ROC analysis performed on the canonical variate (CanVar1) gave rise to a statistically significant discrimination (AUROC = 0.86, p < 0.0001) and maximal sensitivity/specificity at cut-off = -0.544 (canonical variate is a z-score with mean zero and unit standard deviation) reaching 89% sensitivity and 71% specificity (**Figure 5B**).

### Non-Specific Predictivity of Cancer Risk With Exosome Concentration and Size

Despite the “non-specific” (no consideration of PSA expression) predictivity is lower than the specific one of PSA-expressing exosomes, it allows for a very considerable predictive power that could be useful for a future ‘first-level screening’ of general cancer risk or cancer staging. To this aim, it is interesting to check the relation structure among non-specific and specific exosome-based biomarkers. The demonstration of a certain degree of independence of ‘aspecific’ (PCnano1, PCnano2) exosome descriptors from PSA specific (NSFC-exo) one, points to the fact cancer-healthy discrimination obtained by exosome size and number builds upon biological features not strictly related to prostate cancer specificity and thus could be used for ‘general’ cancer screening.

**Table 4** reports the pairwise correlation between the above mentioned aspecific and specific scores. The specific biomarker (NSFC-exo) only has a statistically significant (but relatively weak) correlation with PCnano2, PCnano1 and PCnano2 are mutually orthogonal by construction and PCnano1 is completely independent (near zero correlation) with NSFC-exo. This result indicates that the ‘cancer-related information’ exploited by both the size and number of exosomes is widely independent to the specific (prostate) cancer type. This result is particularly promising for the future use of size and concentration of exosomes as a general cancer biomarker.

**Table 4:** Pearson correlation coefficients between PCnano1, PCnano2 and NSFC exo.

Pearson correlation coefficientsProb > |r| under H_0_: Rho=0Number of observations			
Variable	PCnano1	PCnano2	NSFC-exo
***PCnano1***	1.00000	0.00000	-0.04868
		1.0000	0.6742
	107	107	77
***PCnano2***	0.00000	1.00000	**0.38477**
	1.0000		**0.0006**
	107	107	77
***NSFC-exo***	-0.04868	0.38477	1.00000
	0.6742	0.0006	
	77	77	77

The bold values show the statistically significant correlations of the specific biomarker (NSFC-exo), since NSFC-exo only has a statistically significant (but relatively weak) correlation with PCnano2.

## Discussion

Although research efforts, the burden of cancer keeps on increasing without showing stop signs or forthcoming hopes that the trend will reverse (45–54). The need to detect new and more effective prevention and therapeutic strategies has been finding promising new hopes in exosomes in recent years because of their unique properties as well as their involvement in several physiological and pathological processes. Previous investigation have shown that these nanovesicles can be purified from body fluids, including plasma, and there characterized and quantified (28, 34, 35). This, study was set up to show through a really objective assay, i.e. Nanoparticle Tracking Analysis (NTA), that physical parameters, such as the number and the size of plasmatic exosomes, could distinguish cancer patients from healthy subjects, with the ultimate goal to provide a new non-invasive tool based on quantification of circulating exosomes for diagnosis and clinical follow up of prostate cancer. We used NTA, a highly reliable and sensitive method of exosomes characterization and quantification (9, 35, 36, 38–40). In this kind of study, when a population of patients with a prostate cancer diagnosis before surgery and medical therapy was compared to a healthy males’group we have been obliged to use “individuals with no signs of urological disease”, as control group; in turn meaning males under 50 years old while the PCa was of over 50 aged males. This allowed us to compare the plasmatic levels of exosomes between healthy males and cancer patients. We preliminary showed that there was no correlation in terms of age between the two groups (**Supplementary Table S1**). Thus, we analysed the data comparing the two groups in term of either number and size. The results showed that plasmatic exosomes from PCa patients were significantly more numerous and smaller as compared to plasmatic exosomes from the group of CTR. Indeed, all analysed variables (concentration, mean, mode, D10, D50, D90) were significantly different between CTR and PCa subjects. Among these non-specific indices, the most discriminating variable was the number of exosomes (p<0.0001). Then, through computing Principal Component Analysis (PCA), we showed that both size (PCnano1) and plasmatic concentration (PCnano2) of exosomes caused significant discrimination between PCa and CTR individuals, but through two independent mechanisms. The mutual independence between size and number of exosomes further was validated through the computation of the canonical variate coefficient. The ROC analysis performed on the combination of the size and number of plasmatic exosomes (canonical variate 1, CanVar1) showed a maximal sensitivity (89%) and specificity (71%) at cut-off = -0.544. This method allows us to discriminate in a statistically significant manner (AUROC = 0.86, p < 0.0001) PCa patients from CTR. Finally, we analyzed the correlation between non-specific and specific exosome-based biomarkers. The specific biomarker based on PSA-expressing exosomes (NSFC-exo) had a statistically significant (but relatively weak) correlation with exosomes number (PCnano2) only, suggesting that the kind of ‘cancer-related’ information provided by both size and number of exosomes is widely independent to the specific (prostate) cancer type. Despite the “non-specific” (no consideration of PSA expression) predictivity is lower than the specific one of PSA-exosome, it allows for a very considerable predictive power that could be useful for a future ‘first-level screening’ of general cancer risk or cancer staging or even in predicting after surgery recurrence.

The “liquid biopsy” based on circulating tumor exosomes is a promising and reliable tool for the diagnosis, monitoring, and prognosis of diseases, including tumors, allowing a better sensitivity and specificity of traditional diagnostic techniques, as well as a reduced use of more invasive methodologies (7, 20, 55–59). A high level of circulating exosomes and their miRNA cargos could be useful as potential diagnostic biomarkers, as was observed for alcoholic hepatitis (60).The enrichment of specific markers makes exosomes valuable tools to investigate new biomarker sources useful for tumor diagnosis and prognosis (1, 5, 18, 26, 31). In men with high PSA levels, exosome gene expression in urine was associated with a better ability to distinguish patients with higher-grade prostate cancer, with the consequent reduction of unnecessary biopsies (61, 62). Based on this scenario, in previous papers, we showed increased plasmatic levels of PSA-expressing exosomes in PCa patients compared to BPH and CTR subjects, supporting the clinical relevance of exosomes as tumor biomarkers (35, 36). These studies have prompted a significant boost regarding the clinical utility of exosomes. We thus focused our attention on physical characteristics of the exosomes, such as their number and size, in order to verify whether they could represent signs of malignancy that allowed to clearly distinguishing the healthy subjects from the tumor patients, regardless to the presence of tumor specific biomarkers, whose identification is of course a primary endpoint (33, 63–66).

Although the existence of an open debate in the extracellular vesicles community (6, 67), there is a common agreement on the use of ultracentrifugation to obtain a the most reliable and useful purification of extracellular vesicles from either cell culture supernatant or body fluids. Thus, we used ultracentrifugation for exosome purification and NTA for quantification and size distribution of exosomes in a plasma volume. As detailed above the results showed that prostate cancer patients had significantly higher exosome levels and a reduced size as compared to healthy individuals, thus supporting the clinical use of this approach and its potential use in screening test for prostate cancer early diagnosis.

In summarizing the novelty of this approach includes: a) the demonstration that the measurement of exosome levels and their size in the plasma of human beings, while apparently aspecific, may be helpful in a ‘general screening’ for the presence of a ‘cancer pathology’. Clearly this is only a ‘preliminary finding’ that in any case represents a warning signal to be followed by more specific investigations; b) the NTA analysis of plasmatic exosomes number and size may be helpful in the follow up of cancer patients underwent either medical or surgical treatment, and we want to emphasize with very reduced costs and no invasiveness, as compared to the current diagnostic equipment.

In conclusion, these results express a high clinical impact, strongly suggesting that the concentration and size of circulating exosomes may implement the equipment of cancer biomarkers, particularly for prostate cancer, thus providing a promising new tool for early-stage cancer detection. The results of our study have shown high level of sensitivity and specificity of both exosome number and size in distinguishing prostate cancer patients from a group of individuals with no sign of urological disease, making this approach potentially useful for screening, diagnosis and follow-up of prostate cancer patients. Accordingly, it is reasonable to speculate to exploit in other cancers the clinical potential of the exosome-based approach with the ultimate and ambitious aim of identifying a universal screening test, which remains currently not available. Furthermore, since resection of the primary tumor has been observed to greatly reduce the level of exosomes in oral cancers (34), and the plasmatic levels of exosomes were related to the presence of a primary tymor, in either melanoma (28) or brain tumors (68), monitoring the number of exosomes could also be a winning strategy to control recurrence following tumor resection and to evaluate the effectiveness of the response to anticancer therapy on the tumor mass. From a pathogenetic point of view it appears highly reasonable that the increased plasmatic levels of exosomes in tumor patients may be due to both the hostile microenvironmental condition, such as acidity (9) and the tumor mass (28, 34). Of course the measurements of exosome plasmatic levels needs a clinical validation in terms of platform technology, but the results of our study strongly support the use of ultracentrifugation and NTA as a reliable technical approach.

## Data Availability Statement

The original contributions presented in the study are included in the article/**Supplementary Material**. Further inquiries can be directed to the corresponding author.

## Ethics Statement

The studies involving human participants were reviewed and approved by Ethics Committee of Istituto Superiore di Sanità (protocol code Rif. Prot. PRE-275/17 on 18/04/2017). The patients/participants provided their written informed consent to participate in this study.

## Author Contributions

ML and SF conceived, designed and supervised the study project. DM and RDR performed the experiments. MM and AS collected blood samples from study participants, written informed consent and relevant clinical information of participants, describing the characteristics of the population studied. DM, ML, AG, RDR, and MM collected the data. AG performed and explained the statistical analysis of the data. DM, ML, AG, and SF wrote the original draft of the manuscript. SF, DM, AG, RDR, and ML revised and edited the last version of the manuscript. All authors contributed to manuscript revision, read, and approved the submitted version.

## Conflict of Interest

The authors declare that the research was conducted in the absence of any commercial or financial relationships that could be construed as a potential conflict of interest.

## Publisher’s Note

All claims expressed in this article are solely those of the authors and do not necessarily represent those of their affiliated organizations, or those of the publisher, the editors and the reviewers. Any product that may be evaluated in this article, or claim that may be made by its manufacturer, is not guaranteed or endorsed by the publisher.

## Abbreviations

CTR, Individuals with no signs of urological disease (Control group); PCa, Prostate Cancer patients; PSA, Prostate Specific Antigen; DRE, Digital Rectal Examination; NSFC-exo, PSA-expressing exosomes analyzed by nanoscale flow-cytometry (NSFC); NTA, Nanoparticle Tracking Analysis; PCA, Principal Component Analysis; PCnano1, exosomes size component; PCnano2, exosomes concentration component; CanVar, Canonical Variate; ROC, Receiver Operating Characteristic; AUC, Area Under the Curve.
