# Supplementary material for: Plasmatic Exosome Number and Size Distinguish Prostate Cancer Patients From Healthy Individuals: A Prospective Clinical Study
Source: Front Oncol. 2021 Oct 20;11:727317. doi: 10.3389/fonc.2021.727317 (PMC8564386; doi:10.3389/fonc.2021.727317)
Supplement: Supplementary file 1 [file DataSheet_1.pdf]

SUPPLEMENTARY MATERIALS

PLASMATIC EXOSOME NUMBER AND SIZE DISTINGUISH PROSTATE CANCER PATIENTS FROM HEALTHY INDIVIDUALS: A PROSPECTIVE CLINICAL STUDY.

Mariantonia Logozzi[1†], Davide Mizzoni[1†], Rossella Di Raimo[1†], Alessandro Giuliani[2], Martina Maggi[3], Alessandro Sciarra[3] and Stefano Fais[1*]

SUPPLEMENTARY TABLE

Supplementary Table S1. Pearson Correlation with Age.

Group	Number (concentration)	Mean (size)	PCnano1	PCnano2
PCa	-0.01 (NS)	-0.02 (NS)	0.06 (NS)	0.04 (NS)
CTR	0.02 (NS)	0.23 (NS)	-0.21 (NS)	-0.20 (NS)

SUPPLEMENTARY FIGURES

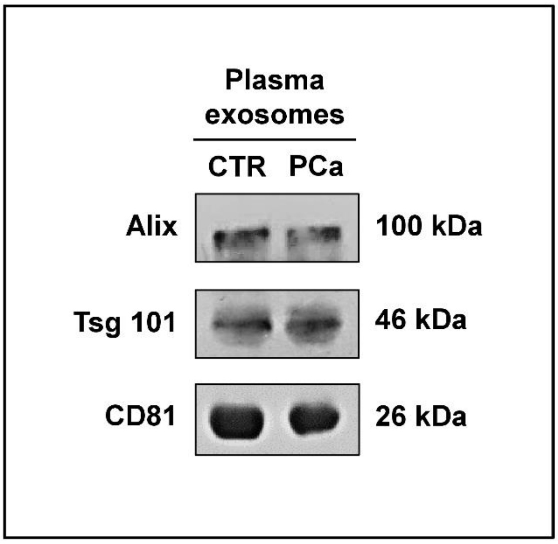

Supplementary Figure S1. Characterization of plasma exosomes from PCA and CTR by Western blot analysis for housekeeping markers. The analyses of anti-Apoptotic linked-gene-product 2 (ALG-2) interacting protein X (Alix, 100 kDa), anti-Tumor susceptibility gene 101 (Tsg 101, 46 kDa), and anti-Cluster of Differentiation 81 (CD81, 26 kDa) monoclonal antibodies was performed on 30 µg of total protein extracts of exosomes purified from 4 ml of pooled plasma of both CTR and PCa patients.

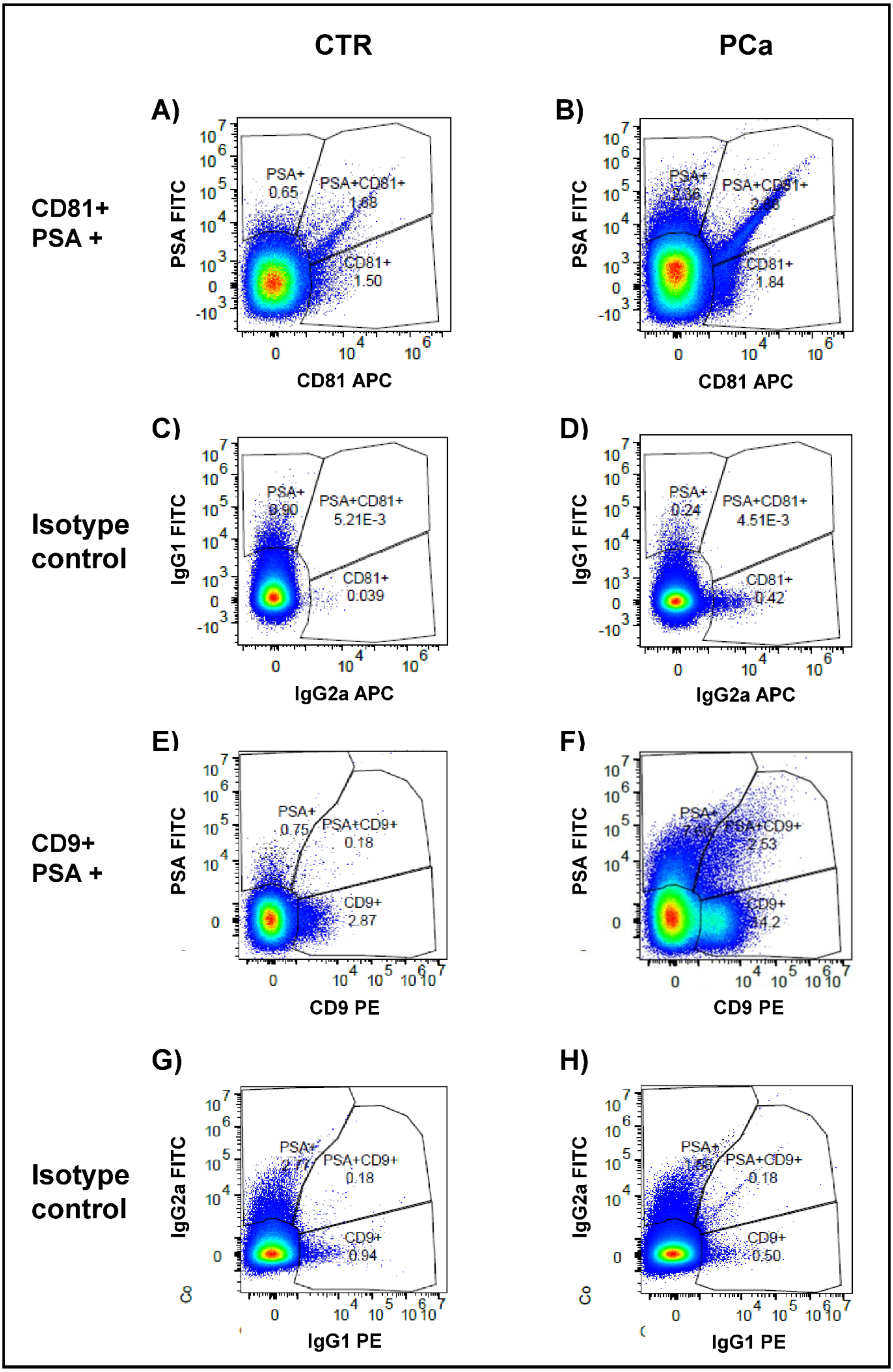
CTR
PCa
CD81+
PSA +
Isotype
control
CD9+
PSA +
Isotype
control
A)
B)
C)
D)
E)
F)
G)
H)
PSA FITC
CD81 APC
PSA+
0.65
PSA+CD81+
1.68
CD81+
1.50
CD81+
1.84
IgG1 FITC
IgG2a APC
PSA+
0.90
PSA+CD81+
5.21E-3
CD81+
0.039
PSA+
0.24
PSA+CD81+
4.51E-3
CD81+
0.42
CD9 PE
PSA+
0.75
PSA+CD9+
0.18
CD9+
2.87
PSA+CD9+
2.53
IgG2a FITC
IgG1 PE
PSA+CD9+
0.18
CD9+
0.94
PSA+CD9+
0.18
CD9+
0.50

Supplementary Figure S2. Characterization of plasma exosomes from PCA and CTR by Nanoscale Flow Cytometry for housekeeping markers. Characterization with anti-human CD81 allophycocyanin (APC) conjugated and anti-human PSA fluorescein (FITC) conjugated of CTR exosomes **(A)** and PCa exosomes **(B)**; anti-human IgG2a (APC) conjugated and anti-human IgG1 (FITC) conjugated were used as isotype control of CTR exosomes **(C)** and PCa exosomes **(D)**, respectively. Characterization with anti-human CD9 phycoerythrin (PE) conjugated and anti-human PSA (FITC) conjugated of CTR exosomes **(E)** and PCa exosomes **(F)**; anti-human IgG1 (PE) conjugated and anti-human IgG2a (FITC) conjugated were used as isotype control of CTR exosomes **(G)** and PCa exosomes **(H)**, respectively.
